# Predictors of very early stroke recurrence in the POINT trial population

**DOI:** 10.1186/s12883-022-02703-4

**Published:** 2022-05-14

**Authors:** Natalie Bourand, James R. Brorson

**Affiliations:** 1 The College, The University of Chicago, Chicago, IL USA; 2 Department of Neurology, MC2030, The University of Chicago, 5841 S. Maryland Avenue, Chicago, IL 60637 USA

**Keywords:** Acute stroke, Ischemic stroke, Secondary prevention, Recurrence

## Abstract

**Background:**

Recent trials of acute secondary prevention in patients with minor ischemic stroke or transient ischemic attack (TIA) have demonstrated high rates of early recurrence within days of the initial event. Identifying clinical features associated with early recurrence may guide focused management.

**Methods:**

Using logistic regression applied to the data of the Platelet Oriented Inhibition in New TIA and Minor Ischemic Stroke (POINT) trial, we evaluated what baseline clinical factors predict outcome events occurring within 7 days of randomization.

**Results:**

In the POINT trial, 181 subjects (3.7%) had early recurrence, defined as primary outcome events within 7 days of trial entry, whereas only 100 outcome events occurred over the remainder of the 90 day trial. Protective effects of dual antiplatelet therapy with clopidogrel plus aspirin were seen only as a reduction in these early recurrences, without any impact on later events. In univariate analysis, systolic blood pressure, diastolic blood pressure, serum glucose, initial carotid imaging results, study cohort (minor stroke or TIA), and treatment assignment were significantly associated with early recurrence. Multivariate logistic regression analysis identified a number of factors with significant independent associations with early recurrence, including carotid stenosis or occlusion (Odds Ratio [OR] 2.77; 95% confidence interval [CI] 1.78–4.31), cohort (minor stroke versus TIA) (OR 1.86; 95% CI 1.33–2.58), race (OR 1.57; 95% CI 1.10–2.25), baseline statin use (OR 0.68; 95% CI 0.49–0.95), systolic blood pressure (OR 1.10; 95% CI 1.03–1.18), serum glucose (OR 1.03; 95% CI 1.01–1.05), and age (OR 1.02; 95% CI 1.00–1.03). Receiver Operator Characteristic (ROC) analysis showed a 70% accuracy of the resulting logistic model in predicting early recurrence.

**Conclusions:**

Early recurrence is high, and is concentrated in the first 7 days, in patients with minor stroke or TIA. A number of baseline clinical factors, including carotid disease, presentation with minor stroke rather than TIA, race, absence of statin usage, systolic blood pressure, and serum glucose, are independently associated with early event recurrence in the POINT trial population.

**Supplementary Information:**

The online version contains supplementary material available at 10.1186/s12883-022-02703-4.

## Introduction

A striking feature of the observations provided by recent trials of acute interventions following minor stroke or transient ischemic attack has been the front-loading of the recurrence events [1]. In the POINT trial, in particular, most of the strokes recorded during the trial’s 90 day follow-up occurred within the first week after randomization [2]. Similar patterns of early recurrence are evident in the published survival curves from other trials of acute interventions for secondary prevention, including the CHANCE, SOCRATES, and THALES trials [3–5]. Analysis of the POINT trial data with kinetic modeling has shown that this characteristic time course can be explained as the result of 2 underlying clinical states, one vulnerable to recurrent ischemia, and one stabilized, with rapid transition from the vulnerable to the stabilized state [6]. Benefits of dual antiplatelet therapy in POINT and CHANCE resulted primarily from prevention of these early recurrence events, rather than by modification of stroke rates over the longer term [7].

Identification of clinical features putting patient at risk for early recurrence after initial minor stroke or TIA is of considerable interest in guiding understanding of mechanisms of stroke recurrence and potentially in directing management in patients at risk. We investigated whether baseline clinical features of subjects in the POINT trial dataset could identify factors placing subjects at risk for early recurrence within 7 days.

## Methods

### Design

Using the data of the POINT trial, we sought to identify baseline clinical factors associated with risk of early recurrence of a secondary event within 7 days following initial minor stroke or TIA. The POINT trial was a prospective clinical, randomized clinical trial in 4881 subjects that compared treatment with aspirin and clopidogrel to aspirin alone in preventing secondary events over 90 days following a TIA or minor ischemic stroke. Subjects were randomized within 12 hours following the incident TIA or minor ischemic stroke. The design, methods, outcome definitions, and results of the POINT trial have been reported previously [8]. The POINT trial dataset was provided by the National Institute of Neurological Disease and Stroke and is available to qualified researchers upon request (https://www.ninds.nih.gov/Current-Research/Research-Funded-NINDS/Clinical-Research/Archived-Clinical-Research-Datasets). Intention-to-treat populations, outcome events, and data censoring methods were defined according to the POINT trial protocol. The University of Chicago Institutional Review Board determined the present study to be exempt from further review.

### Statistical analyses

Subjects with primary outcome events (ischemic stroke, myocardial infarction, or ischemic vascular death) occurring within 7 days of enrollment were categorized in the early recurrence group, and all other subjects, including censored subjects, as those without early recurrence. Kaplan-Meier survivor function analyses were performed using SAS Studio® version 9.4 (SAS Institute, Cary, NC), using the “LIFETEST” procedure with right-sided censoring, separately on subjects with early recurrence and on subjects without early recurrence, in each case censoring all subjects in the other group.

In univariate analysis, a broad set of baseline subject characteristics recorded in the POINT trial dataset were compared between subjects with early recurrence and those without, using R Studio software Version 1.2.5033. Continuous variables were evaluated using the Student t-test if normally distributed, and with the Mann-Whitney U-test if non-normally distributed. Categorical variables were compared using Fisher’s Exact Test or the Chi-Squared Test. A *p*-value of less than 0.05 was considered significant, and a value of less than 0.2 as showing a trend towards significance.

For the multivariate logistic regression analysis, the relevant categorical variables (race, carotid imaging results) from the statistically significant univariate analysis outcome were transformed into binary quantities, with any missing data encoded with “0”. Continuous variables were used directly, except for the category of age, where values encoded as “> 89” were converted to “90”. Results for Race were dichotomized, assigning “1” for Black/African American and Indian/Alaskan Native, categories associated with increased risk of early recurrence in univariate analysis, and “0” for all other categories. For carotid imaging data, “0” encoded for no occlusion, stenosis at < 49%, or missing values, whereas “1” was for occlusion or any stenosis over 50%, on either right or left side. Multivariate logistic regression models, including factors found to have trends towards significance in univariate analysis, were applied using SAS Studio®.

The predictive accuracy of the logistic regression results was assessed with Receiver Operating Characteristic (ROC) analysis. The parameters obtained in logistic regression models were used to calculate a predicted log odds ratio for early recurrence for each subject, which was then compared to the actual outcomes.

## Results

Of 4881 enrolled subjects in the POINT dataset, 181 (3.7%) reached the primary trial outcome of ischemic stroke, myocardial infarction, or death within 7 days of enrollment. Only 100 outcome events occurred in the remainder of the 90 day trial period. The great majority of the early recurrence events (175) were ischemic strokes; 8 were myocardial infarctions (2 with ischemic stroke also), and none were deaths. Early recurrences occurred in 111 subjects in the control group, treated with aspirin alone, and in 70 in the group treated with aspirin and clopidogrel. Only 8 major hemorrhages, the primary safety event, occurred within 7 days.

**Fig. 1:**
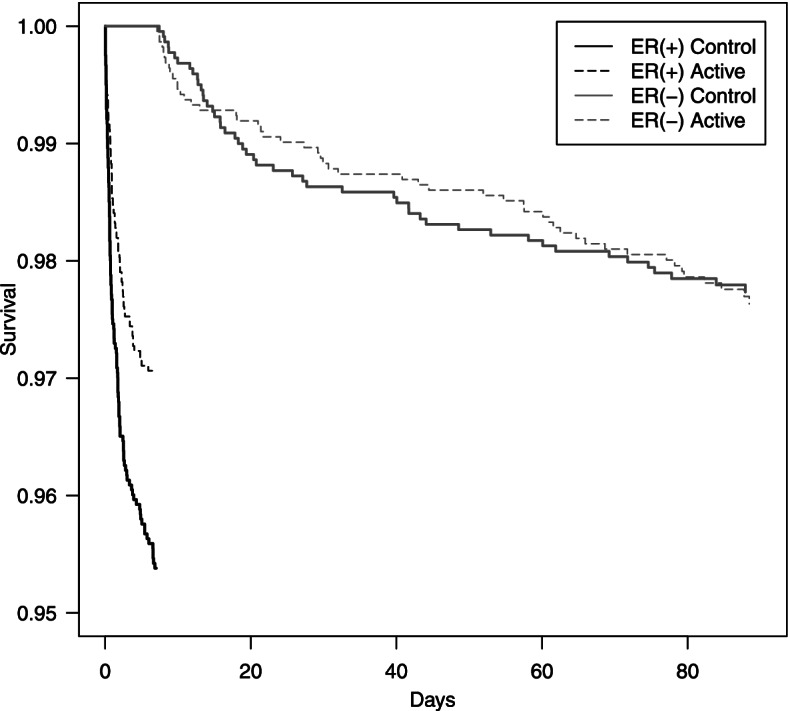
Separate Kaplan-Meier survivor function estimates for POINT trial subjects with early recurrence and those without early recurrence, designated as ER(+) and ER(−), respectively, for active treatment (clopidogrel + aspirin) and control (aspirin) groups. Survival free from the composite primary endpoint of ischemic stroke, myocardial infarction, or death is plotted versus time from study entry, within 12 hours of the initial minor stroke or TIA. A significant treatment effect was found in the subjects with early recurrence, but not with those without early recurrence

An analysis of survival free of outcome events separating subjects with early recurrence from those without early recurrence shows a rapid event rate over the first 7 days compared to a strikingly lower rate of events over the remainder of the 90 day study period (Fig. 1). In the subjects without early recurrence, 49 events occurred in the control group and 51 events in the group treated with aspirin plus clopidogrel. In Kaplan-Meier analyses, the protective effect of dual antiplatelet therapy was confined to the subgroup with early recurrence (Wilcoxon Chi-squared 9.25, 1 df, *p* < 0.01), without any significant difference between treatment groups in the remainder of the 90 day study period in the subgroup without early recurrence (Wilcoxon Chi-squared 0.008, 1 df, *p* = 0.93). We therefore asked which baseline features are associated with this risk of early recurrence, and whether these factors might allow a prior identification of this subgroup bearing most of the risk of recurrent events, and benefitting most from intervention with dual antiplatelet therapy.

A number of clinical baseline features that might relate to risk of early recurrence were examined in univariate analysis, comparing patients with early recurrence to those without. Patients in the group with early recurrence differed significantly from other patients only in cohort (with the index event more likely to be minor stroke than TIA), serum glucose, systolic blood pressure, diastolic blood pressure, and, as expected, by assigned treatment (Table 1). Carotid imaging results showing stenosis > 50%, findings often available soon after initial presentation, were also strongly associated with early recurrence. Several other potential predictors, including age, race, history of hypertension or peptic ulcer disease, and treatment with statin medications, showed trends towards differences in univariate analysis.

**Table 1:** Predictors of Early Recurrence (ER) in the POINT trial population - Univariate analysis

Predictor	ER (−) (***N*** = 4700)	ER (+) (***N*** = 181)	P
Demographics			
Age (median, yrs) [IQR]	65 [55,74]	64 [57,76]	0.19^a^
Gender (% male)	55.0	55.8	0.89 ^b^
Race (% white)	73.1	65.2	0.11^c^
Ethnicity (% Hispanic or Latino)	8.0	6.1	0.62^b^
Medical history			
Hypertension (%)	68.8	76.2	0.076^c^
Diabetes Mellitus (%)	27.3	48.4	0.27^c^
Smoking status (% present smoker)	20.5	24.3	0.40^c^
Ischemic heart disease (%)	10.3	7.7	0.23^c^
Carotid disease (%)	4.2	5.5	0.44^c^
Congestive heart failure (%)	2.6	1.1	0.49^c^
Valvular heart disease (%)	1.7	2.2	0.71^c^
Atrial fibrillation (%)	1.0	1.1	0.83^c^
Peptic ulcer disease (%)	1.0	0.6	0.080^c^
Medications at baseline			
Aspirin (%)	57.6	59.7	0.63^b^
Statins (%)	39.0	32.0	0.13^c^
Other lipid lowering agents (%)	4.8	2.8	0.28^c^
Clopidogrel (%)	1.9	1.1	0.78^c^
Dipyridamole (%)	0.4	0.0	1^c^
Ticlopidine (%)	0.0	0.0	1^c^
Anticoagulants^c^ (%)	3.3	3.3	1^c^
Other interacting agents (%)	21.1	19.3	0.67^c^
Clinical features at presentation			
Cohort (minor stroke vs. TIA) (% minor stroke)	56.3	70.7	< 0.001^b^
Glucose (mg/dL) (median) [IQR]	110 [97, 138]	121 [105, 155]	< 0.001^a^
WBC (10^3^/uL) (median) [IQR]	7.4 [6.1, 8.9]	7.3 [5.9, 9.3]	0.96^a^
RBC (10^6^/uL) (median) [IQR]	4.7 [4.3, 5.0]	4.7 [4.3, 5.1]	0.42^a^
Hgb (mg/dL) (median) [IQR]	14.1 [13.1, 15.1]	14.2 [13.1, 15.1]	0.75^a^
Hematocrit (%) (median) [IQR]	42.0 [39, 45]	42.3 [39, 45]	0.64^a^
Platelets (10^3^/uL) (median) [IQR]	227 [190, 270]	233 [190, 266]	0.90^a^
Systolic BP (mmHg) (median) [IQR]	158 [143, 179]	168 [150, 193]	< 0.001^a^
Diastolic BP (mmHg) (median) [IQR]	87 [76, 98]	90 [80, 104]	0.0042^a^
Treatment			
Arrival to randomization time (min) (median), [IQR]	− 251 [− 375, − 167]	− 225 [− 384, − 176]	0.38^a^
Treatment assignment (%)	49.7	61.3	0.0029 ^b^
Clinical findings			
Carotid imaging results (% with any stenosis > 50%)	3.6	8.2	< 0.001^c^

^a^ By Wilcoxon Rank Sum test; ^b^ by Chi-squared testing with Yate’s correction; ^c^ By Fisher’s Exact test

To evaluate which of these clinical features were independent predictors of early recurrence, multivariate logistic regression was applied. An initial model (Model A) was constructed that consisted only of the immediately identifiable baseline predictors with significant or near-significant differences (*p* < 0.08) in univariate analysis, including cohort, history of hypertension, baseline glucose, systolic blood pressure, diastolic blood pressure, and treatment assignment. A second model (Model B) additionally included other predictors with trends towards significance in univariate analysis, consisting of all factors in Model A plus age, race, history of peptic ulcer, statin use at baseline, as well as carotid imaging results showing any significant stenosis or occlusion, a result often available soon after initial presentation.

**Fig. 2:**
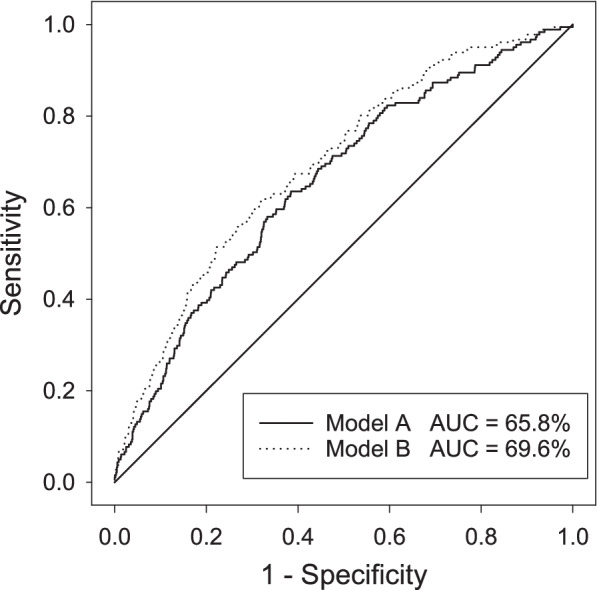
Receiver Operating Characteristic (ROC) analysis of logistic regression models of prediction of early recurrence in the POINT trial. Results are shown for Model A, including predictors with *p* < 0.08 in initial univariate analysis, and Model B, including all predictors with *p* < 0.20 in initial univariate analysis

Logistic regression analysis showed that in Model A, cohort (minor stroke versus TIA), serum glucose, systolic blood pressure, and treatment assignment (aspirin versus aspirin plus clopidogrel) were independent predictors of early recurrence, while in Model B, cohort, systolic blood pressure, serum glucose, age, race, absence of statin treatment, carotid imaging, and treatment assignment were independent significant predictors of early recurrence (Table 2). Carotid imaging results showing significant stenosis or occlusion (OR 2.77), Cohort of minor stroke rather than TIA (OR 1.86), African-American or Indian/Native Alaskan race (O.R. 1.57) and, inversely, prior statin use (O.R. 0.68) were particularly strong predictors of risk of early recurrence, in addition to the expected strong influence of treatment assignment (O.R. 0.62). Model A produced concordant prediction of outcome in 65.8% of the data set (Wald Chi -square statistic 56.7 with 6 df, *p* < 0.0001), while Model B increased the concordant prediction to 69.6% (Wald Chi-squared statistic 90.3 with 11 df, *p* < 0.0001). ROC analysis (Fig. 2) demonstrated moderate predictive strength for Model B, with an odds ratio cut-off of 0.03 producing a sensitivity of 73% and specificity of 53% in prediction of early recurrence.

**Table 2:** Results of Logistic regression analysis for Models A and B

Predictor	Model A		Model B	
	Beta (+/− S.E.)	O.R. [95% limits]	Beta (+/− S.E.)	O.R. [95% limits]
Intercept	−5.89 +/− 0.49**	–	−6.86 +/− 0.71**	–
Hypertension hx	0.20 +/− 0.18	1.22 [0.85, 1.74]	0.118 +/− 0.190	1.13 [0.78, 1.63]
Glucose (per 10 mg/dL)	0.023 +/− 0.010*	1.02 [1.00, 1.04]	0.027+/− 0.010**	1.03 [1.01, 1.05]
Systolic BP (per 10 mmHg)	0.117 +/− 0.033**	1.12 [1.05, 1.20]	0.098 +/− 0.034**	1.10 [1.03, 1.18]
Diastolic BP (per 10 mmHg)	0.004 +/− 0.052	1.00 [0.91, 1.11]	0.028 +/− 0.056	1.03 [0.92, 1.15]
Cohort	0.62 +/− 0.17**	1.86 [1.34, 2.58]	0.61 +/− 0.19**	1.86 [1.33, 2.58]
PUD history	–	–	− 0.55 +/− 1.02	0.58 [0.08, 4.27]
Age	–	–	0.016 +/− 0.007*	1.02 [1.00, 1.03]
Race^a^	–	–	0.45 +/− 0.18*	1.57 [1.10, 2.25]
Statin use	–	–	−0.38 +/− 0.17*	0.68 [0.49, 0.95]
Carotid imaging results^b^	–	–	1.02 +/− 0.26**	2.77 [1.78, 4.31]
Treatment	−0.49 +/− 0.16**	0.62 [0.45, 0.84]	−0.47 +/− 0.16**	0.62 [0.46, 0.85]

* *p* < 0.05; **p < 0.01

^a^Identified as Black/African-American or American Indian/Alaskan Native

^b^Maximum carotid stenosis of > 50% or occluded

## Discussion

The POINT trial clearly demonstrated that the risk of recurrence of stroke following an index minor stroke or TIA was highest in the first several days, and that rapid initiation of dual antiplatelet therapy significantly reduced this risk over the 90 day trial period [8]. Furthermore, subsequent time-based analysis showed that the benefits of dual antiplatelet therapy largely accrued over the first 21 days of treatment, rather than over the entire trial period [2]. The present analysis suggests further that the benefit of dual antiplatelet therapy may actually be confined to prevention of the very early recurrences within the first 7 days after initial stroke or TIA. The overall rate of early 7-day recurrence captured in the POINT trial, in which careful observation of subjects started at enrollment within 12 hours of index TIA or stroke, was 3.7%. This exceeds estimates of early risk from some previous reports, such as the 7-day risk of 1.9% following first-ever TIA [1] and the low risk of 0.8% for in-hospital recurrence following TIA or stroke over a 5-day median duration hospital stay [9]. Thus these very early recurrence events are of particular interest both as a substantial threat to patient stability warranting monitoring and the effective target of enhanced antiplatelet therapy. We sought to examine which clinical factors present at baseline are associated with these early recurrences.

Several baseline factors showed significant associations with early recurrence. Specifically, clinical cohort, systolic blood pressure, serum glucose, age, race, statin use, carotid stenosis, and treatment assignment were independent predictors of early recurrence in multivariate logistic regression analysis. In particular, significant carotid disease with stenosis > 50% or occlusion produced a nearly 3-fold increase in risk, having minor stroke rather than TIA as the index event nearly doubled the risk, and black/African American or American Indian/Alaskan Native race increased the risk 1.57-fold. Prior treatment with statin strongly reduced risk of early recurrence. As expected, treatment with dual antiplatelet therapy also produced a strong protection against early recurrence, with odds ratio 0.62 for early recurrence as compared to aspirin alone.

Some of these factors have been identified previously as predictors of early recurrence following ischemic stroke. Symptomatic carotid stenosis is known to carry a front-loaded risk of stroke following presentation with initial minor stroke or TIA, and is independently associated with in-hospital stroke recurrence [9, 10]. Prior analysis of POINT trial data for predictors of outcome events has shown that black race is significantly associated with recurrences over the entire 90 day study period [11], concordant with the present findings that race can predict very early recurrences within 7 days. Prior statin use has been shown to be associated with lesser early mortality following ischemic stroke [12], and the present results support the prevention of early recurrences as one of the likely reasons. Other factors known to increase risk for long-term stroke recurrence, including age, systolic blood pressure, and serum glucose appear in the present results to also pose risks for very early recurrence.

With regard to cohort, Erdur et al. [9] found that initial TIA rather than stroke was associated with greater risk of in-hospital recurrence over a median hospital stay of 5 days. This contrasts to the results of the present study showing a substantially greater risk of recurrence in the subjects with minor stroke rather than TIA as the entry event in the POINT trial population. The two studies differed in the definition used for qualifying TIA events, with the prior study employing a time -based definition of TIA, independent of imaging findings, while in the POINT trial cases with reversal of signs but positive imaging evidence for infarction were classified as strokes. However, this does not account for the contrasting results, as a re-analysis of the POINT trial data using a time-based rather than imaging-based definition of TIA showed that Cohort remained a strong independent predictor of early recurrence (O.R. 1.81, 95% C.I. 1.32–2.48), with very similar predictive strengths of the Models A and B (See Supplemental data). Thus there is robust evidence that in the POINT trial population minor stroke confers higher risk for early recurrence than does TIA, whether defined based on duration of symptoms or on imaging results.

The logistic regression results provide for the estimation of the odds ratio of early recurrence based on the clinical predictors. The present analysis shows a modest predictive power of such prediction, with concordance rate of about 70% and, using an odds ratio cut-off of 0.03, a sensitivity of 73% and specificity of 53%. Unfortunately this model falls short of the sensitivity required for clinical application, allowing highly reliable identification of the proportion of cases unlikely to go on to early recurrence, who might be assigned to a lower level of care intensity. These baseline clinical features alone are not sufficient to reliably identify patients with minor stroke or TIA who will suffer early recurrence. This concords with clinical experience, in which the occasional sudden deterioration of a stroke patient who is initially mildly affected can come as an unanticipated disappointment.

Other studies have examined factors predictive of event recurrence in stroke or TIA patients [9, 13, 14], generally looking at outcome events over longer time periods than the 7 day period that is the focus of the present study. Most studies have supported the front-loading of recurrences to the early days and weeks following the initial event. Ay et al. [13], constructing an estimator for secondary outcome event over 90 days following incident stroke that they termed the RRE-90, found that history of recent TIA/stroke was a significant clinical predictor of recurrence, but found no evidence for conventional risk factors like history of smoking, hypertension, or diabetes mellitus conferring risk. Lemmens et al. [14] in providing an overview of the various scores for risk prediction following TIA or stroke, found the ABCD2 score to be the best predictor of recurrent stroke after TIA, and the RRE-90 to show potential for predicting risk after stroke, with optimal predictions at 90 day windows. However none of the predictive models reached levels of high concordance with outcome sufficient to be applied for reliable clinical application to decisions regarding individual patients [14]. It seems that both for prediction of very early recurrence and for that of recurrences over 90 days, better markers of risk need to be identified.

Some practical implications for clinical management can be considered from this analysis. In addition to supporting application of the findings of the POINT trial with rapid initiation of dual antiplatelet therapy in qualifying patients, the present results corroborate previous studies that suggest a protective effect of statin treatment against early stroke recurrence [12, 15]. Elevated serum glucose levels and elevated systolic blood pressure show independent association with early recurrence, highlighting these factors as potential targets for acute secondary prevention efforts.

This study has some limitations. First, the proportion of subjects in the POINT trial suffering early recurrence was relatively low, so that the logistic regression is based on the rather small number of patients positive for the outcome of interest, limiting the statistical power of the analysis. Second, these results are based on a select group of patients, meeting the strict inclusion and exclusion criteria of the POINT trial. The results need to be reproduced and extended on a wider group of patients similarly presenting with minor stroke or TIA. Nevertheless, the POINT trial population reflects a common presenting clinical scenario in stroke treatment settings, and the present results identify several factors as independent predictors of early recurrence.

## Conclusions

Recognition of the substantial risk of early recurrence of stroke in the immediate days following an initial minor stroke and TIA should serve as a motivation for rapid application of measures for secondary prevention that may avert such early recurrences [16]. Beyond the use of immediate treatment with dual antiplatelet agents, as supported by the results of the POINT trial and other recent trials, limited observational or prospective trial evidence supports the practices of in-hospital initiation or resumption of statin therapy in ischemic stroke patients [12, 15, 17], and of early intervention (within 2 weeks) for revascularization in patients with symptomatic ipsilateral high-grade internal carotid artery stenosis [10, 17]. The present results underscore the importance of acute management interventions directed at prevention of secondary events, and highlight the treatment of ischemic stroke as a time-critical endeavor [16].

## Supplementary Information

**Additional file 1.**

## Data Availability

The POINT trial dataset is available to qualified researchers through the Archive of Clinical Research Data of the National Institute of Neurological Disease and Stroke (https://www.ninds.nih.gov/Current-Research/Research-Funded-NINDS/Clinical-Research/Archived-Clinical-Research-Datasets). Requests can be made to the NINDS Clinical Research Liaison as described at that website. Details of statistical data from the present study are available by request to the corresponding author.

## Abbreviations

BP: Blood pressure
CI: Confidence interval
ER: Early recurrence
IQR: Interquartile range
OR: Odds ratio
ROC: Receiver operating characteristic
SE: Standard error
TIA: Transient ischemic attack
