# Supplementary material for: Predictors of very early stroke recurrence in the POINT trial population
Source: BMC Neurol. 2022 May 14;22:177. doi: 10.1186/s12883-022-02703-4 (PMC9107185; doi:10.1186/s12883-022-02703-4)

**Supplemental Data.**

Exploratory analysis of the POINT trial data was conducted re-defining ‘Cohort’ (minor stroke versus TIA) using a time-based definition of TIA, rather than imaging- based definition. All cases with symptoms not persisting at the time of randomization and symptom duration of less than 24 hours, regardless of imaging results, were classified as time-based TIAs, This definition produced reclassification of 315 cases from minor stroke to TIA, and of 1 case from TIA to minor stroke.

Using this time-based TIA definition, in univariate analysis, 49.8% of ER(-) events were minor stroke, while 64.6% of ER(+) cases were minor stroke, a difference significant by Chi-squared testing (p< 0.0001).

Logistic regression analyses using Model A and Model B as previously defined, were conducted with substitution of the time-based determination of Cohort replacing the imaging-based determination (Table). The predictors showing independent association with early recurrence were unchanged.

**Table.** Results of Logistic regression analysis for Models A and B, using Cohort assignment employing a time-based definition of TIA.

| Predictor | Model A |  | Model B |  |
| --- | --- | --- | --- | --- |
|  | Beta (+/- S.E.) | O.R. [95% limits] | Beta (+/- S.E.) | O.R. [95% limits] |
| Intercept | -5.81 +/- 0.49** | - | -6.75 +/- 0.70** | - |
| Hypertension hx | 0.19 +/- 0.18 | 1.22 [0.85, 1.74] | 0.118 +/- 0.190 | 1.13 [0.78, 1.63] |
| Glucose (per 10 mg/dL) | 0.023 +/- 0.010* | 1.02 [1.00, 1.04] | 0.026+/- 0.010** | 1.03 [1.01, 1.05] |
| Systolic BP (per 10 mm Hg) | 0.116 +/- 0.033** | 1.12 [1.05, 1.20] | 0.098 +/- 0.034** | 1.10 [1.03, 1.18] |
| Diastolic BP (per 10 mm Hg) | 0.005 +/- 0.052 | 1.00 [0.91, 1.11] | 0.028 +/- 0.056 | 1.03 [0.92, 1.15] |
| Time-based Cohort | 0.59 +/- 0.16** | 1.86 [1.34, 2.58] | 0.59 +/- 0.16** | 1.81 [1.32, 2.48] |
| PUD history | - | - | -0.58 +/- 1.02 | 0.56 [0.08, 4.15] |
| Age | - | - | 0.016 +/- 0.007* | 1.02 [1.00, 1.03] |
| Race^1^ | - | - | 0.45 +/- 0.18* | 1.57 [1.10, 2.25] |
| Statin use | - | - | -0.38 +/- 0.17* | 0.68 [0.49, 0.95] |
| Carotid imaging results^2^ | - | - | 1.04 +/- 0.22** | 2.84 [1.83, 4.41] |
| Treatment | -0.49 +/- 0.16** | 0.62 [0.45, 0.84] | -0.48 +/- 0.16** | 0.62 [0.46, 0.85] |

* p < 0.05; **p < 0.01

Receiver operator characteristic (ROC) analyses for Model A and Model B, based on time-based Cohort determination, were conducted, producing areas under the curve (AUC) results of 0.657 and 0.697, respectively, for Model A and Model B (Figure).

Supplemental Figure : A) ROC analysis for Model A, using time-based definition of TIA. B) ROC analysis for Model B, using a time-based definition of TIA.

A


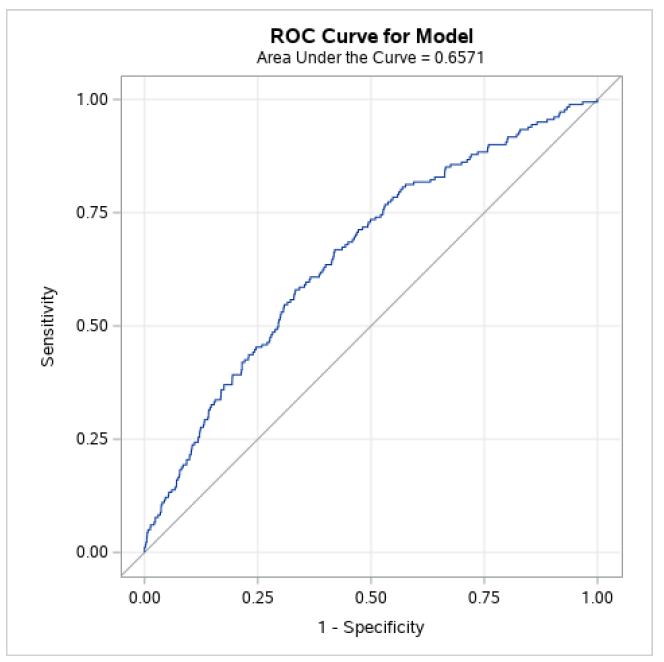


B


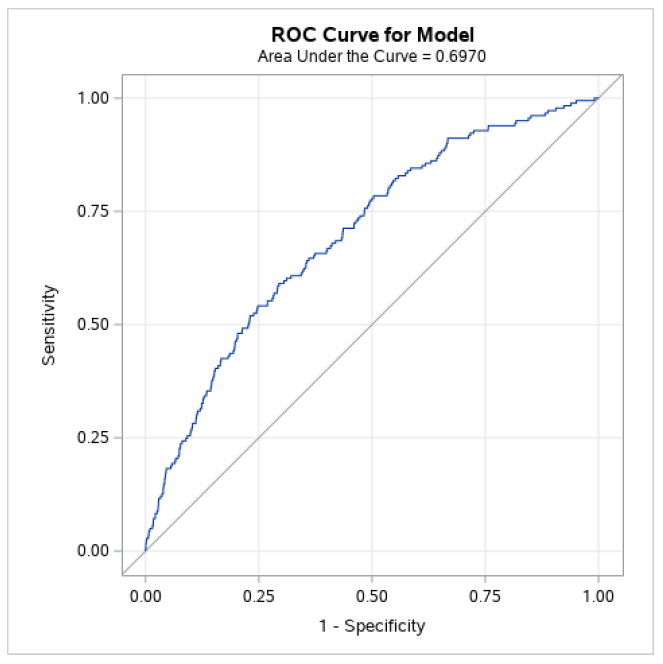

Supplement: Supplementary file 1 — Additional file 1. [file 12883_2022_2703_MOESM1_ESM.docx]
